# Supplementary material for: Taxonomy of the Genus Halophila Thouars (Hydocharitaceae): A Review
Source: Plants (Basel). 2020 Dec 8;9(12):1732. doi: 10.3390/plants9121732 (PMC7763217; doi:10.3390/plants9121732)
Supplement: Supplementary file 1 [file plants-09-01732-s001.zip › Supplementary-plants-990190/Supplementary A Final.pdf]

Supplementary A

Halophila Specimens Studied from the Following Herbaria

Herbarium		Specimen No.
AD	South Australia State Herbarium	89
APF	Universidale de Sao Paulo, Brazil	7
BISH	Bishop Museum & Herbarium	49
BO	Herbarium Bogoriensis, Indonesia	35
BSI	Botanical Survey of India	25
C	Copenhagen Botanical Garden Herbarium	82
CAN	Canberra National Herbarium	6
DNA	Northern Territory Herbarium, Darwin	5
F	Philippine Univ., Marine Science Institute Herbarium	30
GU	Guam University Herbarium	34
HO	Tasmania, Hobart Herbarium	8
HW	The Hebrew University of Jerusalem	13
K	Kew Botanical Garden Herbarium	115
KL	Kasetasrt University, Thailand	13
KYO	University of Kyoto Herbarium, Japan	8
LA	Larkum Collection , Sydney	27
MOZ	Univesiasae Eduardo Mondlane Herbarium	19
NHB	British Natural History Museum	122
L	Leiden Herbarium	184
MEL	Victoria National Herbarium	56
MELU	University of Melbourne	26
NSM	National Science Museum, Tokyo	15
NSW	New South Wales State Herbarium	90
NY	New York Botanical Garden Herbarium	75
PERTH	Western Australia Herbarium, Perth	49
PRE	National Herbarium Pretoria, South Africa	23
QFH	Queensland Fishery Herbarium	30
QH	Queensland State Herbarium	47
S	Naturhistoriska Riksmuseet Stockholm, Sweden	27
SBG	Singapore Botanical Garden Herbarium	39
SS	Selmar Schonland, South Africa	20
TNS	Tokyo National Science Herbarium	17
UC	University of California, Berkeley	202
UM	University of Malaysia	14
US	Smithsonian Institute Herbarium	107
USF	University of South Florida	41
UT	University of Tokyo Herbarium	23
UWA	University WA Botany Herbarium	230
W	Vienna National Museum	41
YLH	Tel Aviv University Herbarium	124
Z	Zurich Botanic Garden Herbarium	35
