# Supplementary material for: Taxonomy of the Genus Halophila Thouars (Hydocharitaceae): A Review
Source: Plants (Basel). 2020 Dec 8;9(12):1732. doi: 10.3390/plants9121732 (PMC7763217; doi:10.3390/plants9121732)
Supplement: Supplementary file 1 [file plants-09-01732-s001.zip › Supplementary-plants-990190/Supplementary B1 Final.pdf]

Supplementary B1

The Locality, Collector and Herbarium Information of Botanical Drawings for the *Halophila* Species

	**Species**	**Locality, collector, herbarium**
Figure S1	*Halophila australis*	South Australian Coast, 14 m, *H. Kirkman* (CSIRO)
		Tasmania Low Head, *F Perrin* (HO)
		Queens Cliff, Victoria, *Lucas 3214* (NSW, type)
Figure S2	*Halophila major*	Malaysia, Johor, *Shafei 1380* (Univ Malaya)
Figure S3	*Halophila ovalis*	Queensland, Green Is., 25m, *R Coles s.n.* (UWA R311, wet)
Figure S4	*Halophila bullosa*	Samoa, *T. Powell* (K)
Figure S5	*Halophila minor*	Indonesia, Java, *HF Neubauer 601* (L)
Figure S6	*Halophila gaudichardii*	Guam, Mona Bay, *B Stone 4063* (L)
Figure S7	*Halophila linearis*	Mozambique, Inhaca Is., *Mogg 29309*, 27730 (L)
Figure S8	*Halophila ramamurthiana*	India, Gujarat, (UWA, dry)
Figure S9	*Halophila mikii*	Japan, Tanegashima, type (KYO)
Figure S10	*Halophila nipponica*	Japan, Chiba, Tateyama, *Y. Hrano s.n.* (UWA R340, 338, wet)
Figure S11	*Halophila okinawensis*	Okinawa, Kin Bay, *Kanamoto s.n.* (UWA R490, wet)
Figure S12	*Halophila hawaiiana*	Hawaii, Oahu, Kahola Beach, *Kanamoto s.n.* (UWA R135, wet)
		Kailua Beach, *FR Forsberg 55603* (US)
Figure S13	*Halophila johnsonii*	Florida, *Moffler 225* (USF)
Figure S14	*Halophila madagascariensis*	Madagascar, type collection (P)
Figure S15	*Halophila stipulacea*	Elate Gulf, *Lipkin 14473* (Tel Aviv Univ.)
Figure S16	*Halophila balfourii*	Mauritius, Grand Bay, *Mortensen s.n.* (BM, C, K)
Figure S17	*Halophila decipiens*	Sydney Harbor, *Brazier & Ramsey 3199* (NSW)
Figure S18	*Halophila capricorni*	Green Is., Queensland (UWA R271, wet)
		Elford Reef, 11m, Lyle (UWA R 261, wet)
Figure S19	*Halophila sulawesii*	Indonesia, Sulawesi Is., *Veheji 417, 542* (L)
Figure S20	*Halophila beccarii*	Trang, Thailand, Kuo (UWA, wet)
Figure S21	*Halophila engelmannii*	Florida, Virginia Key, *Hawe 125* (NY)
Figure S22	*Halophila baillonis*	Proto Rico, *Howe 221* (C, NY)
Figure S23	*Halophila spinulosa*	Carnarvon, WA, Australia, *Ostenfeld 274* (C, NY)
Figure S24	*Halophila tricostata*	Queensland, Corwie Pt. 31 m, (QFH 67 & UWA R374, wet)

Note that Figure S1, Figure S12 and Figure S18 have used more than one specimen.

Legend applies to all botanical drawings (Figures S1-S24).

**A**: anther	**ES**: Extended Shoot	**FF**: Female Flower	**Fr**: Fruit
**MF**: Male flower	**P**: Petiole	**PS**: Petiole Scale	**RM**: Rhizome
**RS**: Rhizome Scale	**St**: Style	**YF**: Young Fruit	
