# Supplementary material for: Taxonomy of the Genus Halophila Thouars (Hydocharitaceae): A Review
Source: Plants (Basel). 2020 Dec 8;9(12):1732. doi: 10.3390/plants9121732 (PMC7763217; doi:10.3390/plants9121732)
Supplement: Supplementary file 1 [file plants-09-01732-s001.zip › Supplementary-plants-990190/Supplementry B2(S1-S24).zip!/Fig S4-6-FINAL.pdf]

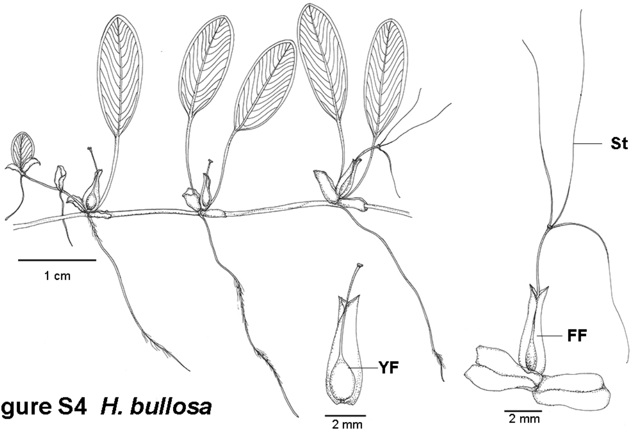

Figure S4 *H. bullosa*

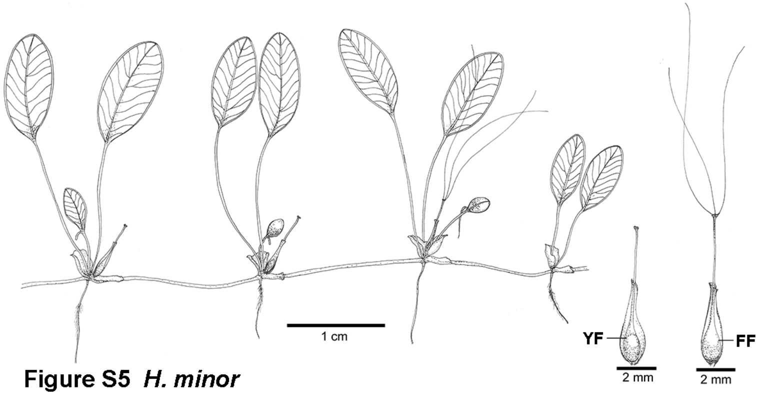

Figure S5 *H. minor*

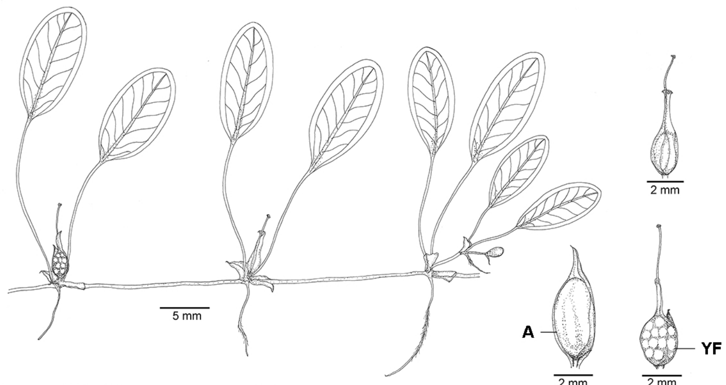

Figure S6 *H. gaudichardii*
